# Optimizing soybean biofuel blends for sustainable urban medium-duty commercial vehicles in India: an AI-driven approach

**DOI:** 10.1007/s11356-024-33210-3

**Published:** 2024-04-23

**Authors:** Upendra Rajak, Prem Kumar Chaurasiya, Tikendra Nath Verma, Abhishek Dasore, Ümit Ağbulut, Kundan Meshram, CAhamed Saleel, Shaik Saboor, Erdem Cuce, Zhibao Mian

**Affiliations:** 1 Department of Mechanical Engineering, RGM College of Engineering and Technology, Nandyal, 518501 India; 2 Department of Mechanical Engineering, National Institute of Technology, Raipur, 492010 India; 3 Department of Mechanical Engineering, Maulana Azad National Institute of Technology, Vellore, 462003 India; 4 Department of Biological and Agricultural Engineering, Faculty of Engineering, Universiti Putra Malaysia, 43400 UPM Serdang, Selangor Malaysia; 5 Department of Mechanical Engineering, Faculty of Mechanical Engineering, Yildiz Technical University, 34349 Istanbul, Turkey; 6 Department of Civil Engineering, Guru Ghasidas Vishwavidyalaya, Bilaspur, India; 7 Department of Mechanical Engineering, College of Engineering, King Khalid University, Abha, 61421 Saudi Arabia; 8 School of Mechanical Engineering, Vellore Institute of Technology Vellore, 632014 Vellore, Tamil Nadu India; 9 Department of Mechanical Engineering, Faculty of Engineering and Architecture, Recep Tayyip Erdogan University, Zihni Derin Campus, 53100 Rize, Turkey; 10 School of Engineering and the Built Environment, Birmingham City University, Birmingham, B4 7XG UK; 11 Faculty of Science and Engineering, School of Computer Science, University of Hull, Cottingham Road, Hull, HU6 7RX UK; 12 Center for Research Impact & Outcome, Chitkara University, 140401 Rajpura, Punjab India

**Keywords:** Artificial intelligence, Diesel engine, Pollutant formation, Soybean biofuel

## Abstract

This article presents the outcomes of a research study focused on optimizing the performance of soybean biofuel blends derived from soybean seeds specifically for urban medium-duty commercial vehicles. The study took into consideration elements such as production capacity, economics and assumed engine characteristics. For the purpose of predicting performance, combustion and emission characteristics, an artificial intelligence approach that has been trained using experimental data is used. At full load, the brake thermal efficiency (BTE) dropped as engine speed increased for biofuel and diesel fuel mixes, but brake-specific fuel consumption (BSFC) increased. The BSFC increased by 11.9% when diesel compared to using biofuel with diesel blends. The mixes cut both maximum cylinder pressure and ${{\text{NO}}}_{{\text{x}}}$ emissions. The biofuel-diesel fuel proved more successful, with maximum reduction of 9.8% and 22.2 at rpm, respectively. The biofuel and diesel blend significantly improved carbon dioxide (${{\text{CO}}}_{2}$) and smoke emissions. The biofuel blends offer significant advantages by decreeing exhaust pollutants and enhancing engine performance.

**Graphical Abstract:**

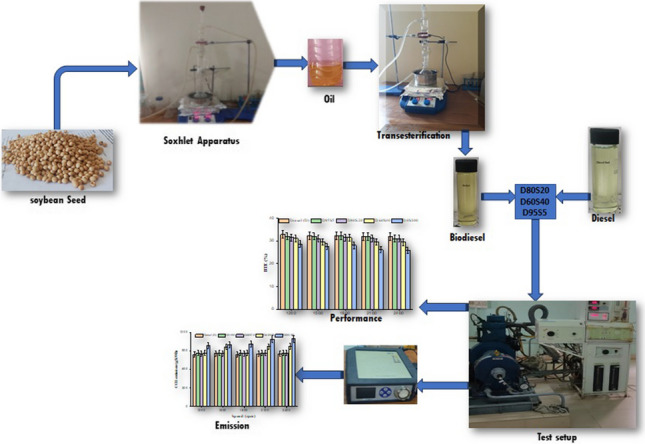

## Introduction

The integration of artificial intelligence (AI) has significantly contributed to the remarkable advancements witnessed in the automotive industry over the past decade. AI has been successfully incorporated into various aspects of vehicle design, operation and optimization. Notably, in the prediction and analysis of engine performance and exhaust emissions, AI plays a pivotal role in enhancing engine efficiency and reducing environmental impacts (Verma et al. 2021a; Dasore et al. 2022). AI offers numerous potential applications, and one area that stands to benefit is the field of diesel engine technology. Diesel engines are generally suitable for heavy-duty applications viz. transportation, agriculture and power generation due to their inherent efficiency and torque characteristics. Diesel engines are commonly associated with emitting higher levels of harmful pollutants such as nitrogen oxides (${{\text{NO}}}_{x}$), particulate matter (PM) and other hazardous substances (Ineza Havugimana et al. 2023).

Conventional approaches of evaluating engine performance, emissions and combustion traits typically rely on time-consuming and costly experimental and computational methods. These methods may have various constraints. Consequently, there is a growing demand for accurate and reliable prediction models that can anticipate engine performance and emissions across different operating conditions (Sharma 2021). Thus, employing AI techniques such as machine learning and neural networks has become a feasible and encouraging alternative approach in modern times. AI models can be trained using historical data from experimental tests to produce accurate predictions regarding engine performance. The specific characteristics include of a vehicle power output, fuel consumption, torque and exhaust emissions, namely carbon monoxide (CO), hydrocarbons (HC), ${{\text{NO}}}_{x}$ and PM (Sharma 2020; Tasdemir et al. 2021).

AI models can be trained to forecast CO, HC, ${{\text{NO}}}_{x}$ and PM emissions. Recently, there has been a strong emphasis on utilizing AI to forecast engine performance, resulting in a substantial amount of study in this field. Researchers have investigated different machine learning and neural network approaches to create precise and dependable models for forecasting engine performance indicators and emissions across a range of operating situations. Table 1 presents a summary of significant research undertaken in this field, together with their main results. The literature assessment confirms that ANN model is highly effective in tackling emission control and performance analysis, producing promising results and achievable benchmarks. AI approaches have demonstrated better results in engine control and diagnosis when compared to fuzzy logic. Advancements in approaches like reinforcement learning and specific algorithms have the potential to improve engine control and diagnosis chores.

**Table 1:** Employing specialized algorithms in reinforcement learning

Research work	Methodology	Key findings
Jiahong et al. (2022)	ANN algorithm	A highly skilled ANN model can accurately forecast engine efficiency and emissions of unburned HC, CO and ${{\text{NO}}}_{x}$ with minimal error
		Root mean square error (RMSE)” the coefficient of determination signifies a significant relationship and suitable inputs and outputs
		Proposes that an ANN model is suitable and efficient for forecasting engine-related variables
		Possibility of enhancing motor development in hybrid automobiles by utilizing a forecasted engine map
	Support vector regression (SVR)	An adept SVR algorithm was trained to accurately predict fuel consumption rate, HC, CO and NO_*x*_ emissions. This was achieved through training. The model coefficient of determination (${R}^{2}$) was closed to one, suggesting a strong correlation between the experimental data and the model predictions
Hao et al. (2018)	Support vector machine (SVM)	The SVM model prediction accuracy is influenced by both the model parameters and the quantity of training samples. Choosing the best SVM regression model and a certain training sample size has significantly improved the expected mean absolute percentage error (MAPE) and maximum relative prediction error (MRE) for ${{\text{NO}}}_{x}$ emission. The errors quantify the model precision in predicting outcomes. The MAPE decreased from 12.54 to 8.32%, and the MRE decreased from 56.6 to 25.89%
Zhang et al. (2022a) Zhang et al. (2022b)	Local distance-based decision trees (LDDTs) and deep learning decision tree CO_2_ emission model (DL-DTCEM)	Two LDDTs were evaluated for on-road ${{\text{CO}}}_{2}$ emissions using a portable emission measuring system (PEMS) and a global positioning system (GPS). The DL-DTCEM ${{\text{CO}}}_{2}$ emissions were developed using deep learning to accurately predict ${{\text{CO}}}_{2}$ emissions from LDDTs. The transient ${{\text{CO}}}_{2}$ emission rate of LDDTs is greatly influenced by the vehicle speed, acceleration, specific power and road slope. The rate of ${{\text{CO}}}_{2}$ emissions was linked to the speed of the vehicles and the incline of the roadways
Kesgin (2004)	Genetic algorithm (GA)	Enhancing engine efficiency while maintaining NO_*x*_ emissions within the required limit of 250 mg/Nm^3^ for stationary engines
		Genetic algorithms and ANN analysis are useful for forecasting and enhancing engine efficiency and NO_*x*_ emissions by considering design and operating data
Warey et al. (2021)	Convolutional neural network (CNN)	The study utilized convolutional neural network (CNN) models to forecast engine out emissions such as CO, HC and smoke emission. The predictions were based on analysing scalar field contours within the cylinder at the exhaust valve opening phase. The scalar field examining this work includes equivalence ratio, temperature, velocity and turbulent energy
Zhang et al. (2022c)	Multistate deep reinforcement learning (M-DRL)	This study presented a novel energy management strategy based on M-DRL with a hybrid action space combining discrete and continuous elements. The state space is expanded to integrate real-time multivariate traffic and terrain information, enhancing the accuracy of the energy management system
Li et al. (2007)	Gaussian process regression (GPR)	GPR model demonstrated exceptional accuracy in predicting performance, temperature and emission values under both steady-state and transient operating conditions, when compared to other regression modeling methods
Wong et al. (2015)	Sparse Bayesian extreme learning machine (SBELM)	In relation to the duration of execution, the size of the model, and its ability to withstand fluctuations in the quantity of hidden neurons, SBELM demonstrates superiority over other approaches. The SBELM model demonstrates its ability to meet the practical criteria of a mathematical engine model, enabling accurate and efficient online engine performance prediction. Its exceptional performance in these areas further supports its effectiveness
Chen et al. (2023)	Embedding graph neural network (EGNN) model united with self-attention mechanism and sensor embedding	EGNN model was developed to effectively capture the intricate relationships within sequences and enhance the capability to accurately predict sequences spanning long time steps
		Overcomes limitations of LSTM and transformer models
Koohfar et al. (2023)	Time series methodologies: traditional (ARIMA and SARIMA) and deep learning (RNN, LSTM and transformers)	Transformer model was employed to forecast EV charging demand. The forecasting encompassed three distinct time steps, namely, 7, 30 and 90 days, effectively addressing both short-term and long-term forecasting requirements. Performance compared using RMSE and MAE. Transformer model outperforms other models in short-term and long-term predictions
Alonso et al. (2007)	Combination of ANNs and GAs	Feasibility study of using ANNs and GAs for diesel engine optimization
		ANNs used as a simulation tool to predict emission levels and fuel consumption based on engine operating parameters
		GA approach used to optimize engine settings based on ANN outputs
Wang et al. (2021)	Blend of dynamic programming and GA	Proposed method for voyage optimization using dynamic programming and genetic algorithm
		Engine power discretized into multiple levels
		Investigated the potential benefits utilizing a medium-size chemical tanker
		Demonstrated fuel-saving and emission reduction compared to deterministic methods
		Average fuel consumption and GHG emission reduction of 5.6% (about 275 tons) compared to full-scale measurements for six case study voyages

In the literature review, it has been established that ANN models exhibit high efficiency in addressing both emission control and performance analysis, yielding promising results and attainable benchmarks. Additionally, compared to fuzzy logic, AI techniques have shown superior outcomes in engine control and diagnosis. To further enhance engine control and diagnosis tasks, there is potential for advancements in techniques such as reinforcement learning and the utilization of specialized algorithms.

This study builds upon previous research regarding the impact of soybean (biofuel) enrichment on a compression direct injection diesel engine running on various biofuel blends (D95S5, D80S20 and D60S40). This study examines the impact of engine speed on performance, combustion and emissions of a direct injection diesel injection by utilizing artificial intelligence to forecast the operational performance and emissions of diesel engines at different speed using a blend of soybean biofuel and diesel fuel. Accurate predictive models can also be utilized to enhance engine design, improving efficiency and environmental impact. Furthermore, precise estimation of exhaust emissions can assist lawmakers in formulating stringent regulations and strategies for pollution control. Indian government is going ahead with a plan to achieve ethanol blending target of 20% with petrol by 2025–2026 and 5% of biodiesel by 2030. Furthermore, the govt. is encouraging for biodiesel production, research and analysis in terms of providing funding resources.

## Material and method

### Fuel and fuel characteristics

**Fig. 1:**
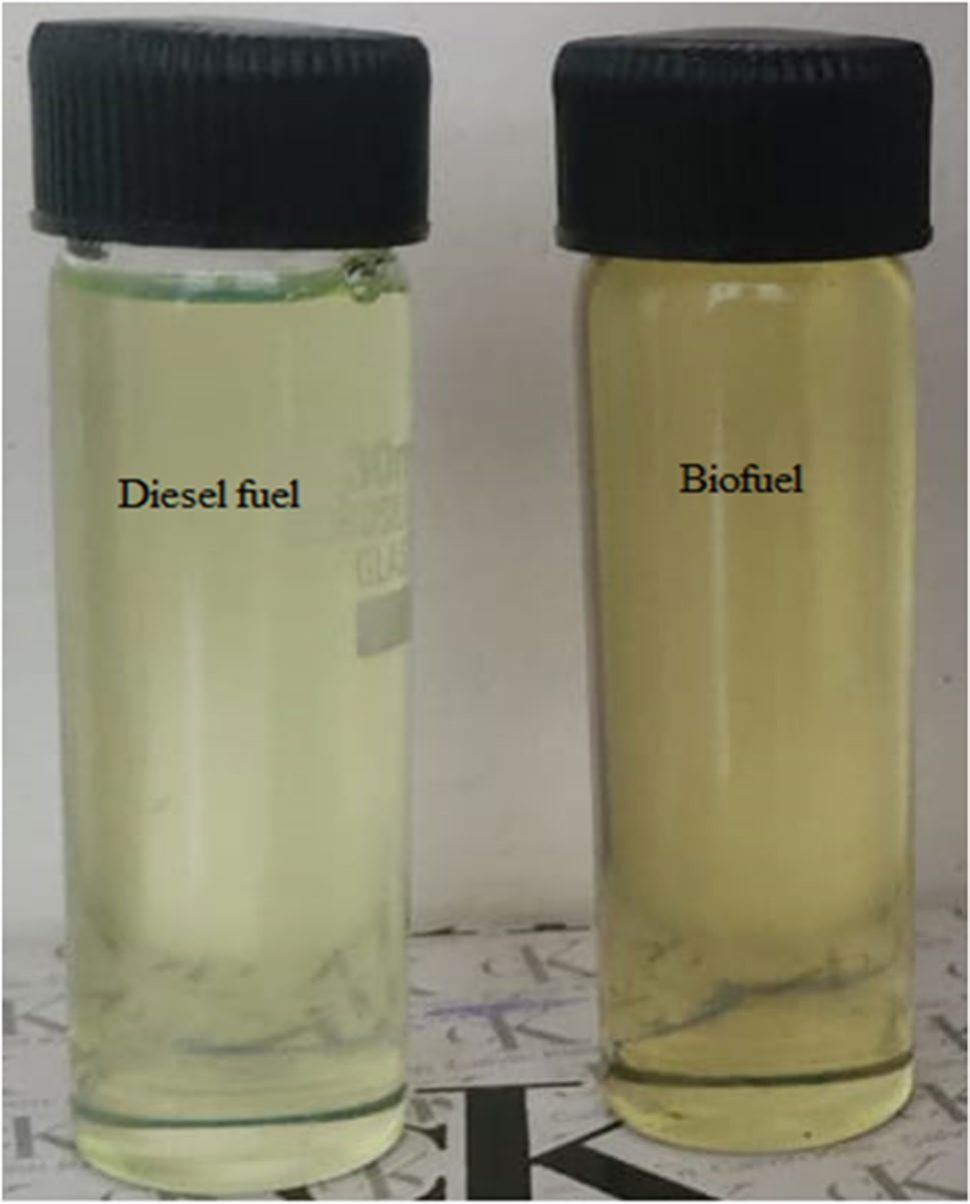
Fuel samples

Only 1% of the world energy comes from biofuels, compared to the 80% that comes from oil and its derivatives. The manufacture of biodiesel has a long history of overcoming challenges (Kiani et al. 2010). This is due to the fact that despite high conversion rates, technologies are still immature and have room for improvement. It is not possible to recycle the homogeneous catalysts that are used in industry, and the methanol that is utilised in the process of transesterification is derived from fossil fuels. Natural resources that are in competition with the food chain are another issue of concern due to the fact that agricultural land is required to produce both energy and sustenance (Prabhu et al. 2023; Aslan 2023). In light of this, one of the continuous issues facing the biodiesel sector is the search for further industrial uses of glycerol, in addition to discovering non-food oil sources that are cheaper, more efficient and provide a larger selection of options. The vast majority of these items are disposed of in ways that are harmful to the environment, such as by dumping them in rivers or landfills (Bibin et al. 2023; Verma 2021b). On the other hand, it is possible to use it in CI engines in lieu of diesel fuel manufactured from petroleum as a straight substitute. For the purpose of this experiment, an S biofuel is being used as the propellant for the vehicle (Fig. 1). Blends of diesel and S biofuel, referred to as “D95S5, D80S20 and D60S40,” include 5%, 20% and 40% biofuel and 95%, 80% and 60% diesel by volume as shown in Table 2.

**Table 2:** Properties of SME

Fuel	D0S100	D60S40	D80S20	D95S5	D (diesel)
Density (g/mL)	0.885	0.852	0.841	0.852	0.830
Kinematic viscosity ($\frac{{mm}^{2}}{s}$)	4.6	3.6	3.44	3.66	3.0
Lower calorific value (MJ/kg)	36.2	39.9	41.2	39.8	42.5
Flash point	120	66	64	63	76
CN	51	49.4	48.6	49.4	48

### Experimental setup

**Fig. 2:**
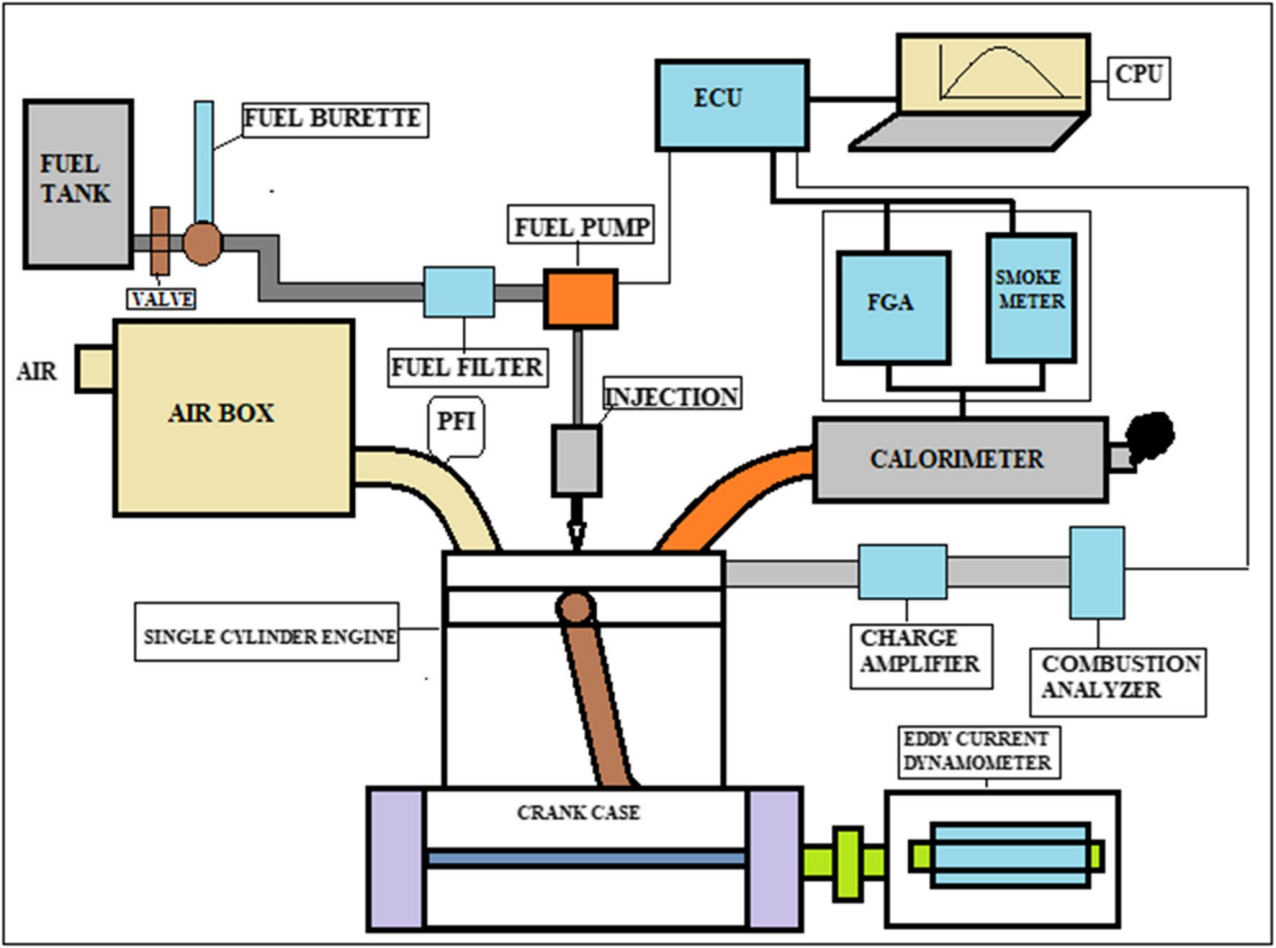
Experimental setup

The experimental work was conducted at the I.C. Engine Laboratory of the RGM College of Engineering and Technology, Nandyal. The tests were conducted with the engine running at variable speeds and under two different loads, with diesel/biofuel mixes D95S5, D80S20 and D60S40, with the injection pump completely open. Diesel (D) and D0S100 were also examined in this study since they demonstrated stabilization under the circumstances that were tested. A common-rail injection system diesel engine with one cylinder, water cooling and four strokes was utilized for testing. The major engine specifications are included in the inventory that can be found in Table 3. Simple tools are used to monitor the engine performance and exhaust emissions under various operating conditions. A commuter equipped with IC ermine software for ignition control offers the ability to analyze engine performance and combustion data from tested samples. This application is used to receive signals for calculating factors such as heat transfer rate, cylinder pressure, fuel flow rate, air flow rate and calorimeter water flow rate. The AVL444 gas analyser is used to monitor ${{\text{CO}}}_{2}$ and ${{\text{NO}}}_{x}$ emissions from the engine tailpipe. The AVL 437C smoke meter is used to measure the concentration of smoke emissions. The instruments used in this testing are regularly calibrated to account for the variability of all parameters. A pictorial representation of the experimental test rig is shown in Fig. 2. A total of 75 tests were carried out with the engine operating at speeds of 1200, 1500, 1800, 2100 and 2400 revolutions per minute. The load cell force values were in kilograms, which corresponded to torque value of 12.5 N.m. The load was stabilised by putting in place an electronic module that continually processed and showed the mean signal value coming from the dynamometer load cell. This was done in order to accomplish load stabilisation. The engine was warmed up for 5 min with the specific blend that was going to be tested before the actual test. Additionally, a cleaning of the supply tank was performed as part of the process of switching fuel blends in order to prevent any changes in the fuel mixes that were being tested. After the exhaust emissions reached a steady state, the data from the test were recorded, and the gas analyser was used to examine them.

**Table 3:** Finding input value for engine

Limits	Limits value
Engine stroke/cylinder	4/1
Injection pressure	Higher than of 230 bar
Speed	1200–2400 rpm
Bore/stroke	87.5/110 mm
Advanced fuel injection timing	24.5° b TDC
Compression ratio	18:1
Method of cooling	Water

### Heat release rate calculation

Since the engine is direct injection, the fuel is the sole mass flowing into the system, its sensitivity enthalpy is near to zero, and the chamber gas is optimal, and $PV=mRT=\frac{{C}_{p}}{{C}_{v}}$ and ${C}_{p}={C}_{v}$ + *R*, and the rate of heat release can be written as Eq. (2) (Zapata-Mina et al. 2023; Ayd 2021).

2 $$\frac{dQ}{d\theta }=\frac{1}{(\gamma -1)}. V \frac{dP}{d\theta }+\frac{\gamma }{(\gamma -1)}.P \frac{dV}{d\theta }$$

where *θ* is the crank angle, *P* is the pressure within the cylinder, $\frac{dQ}{d\theta }$ is the rate at which heat is released, *V* is the fluctuation in volume in relation to and is the cause for the particular temperatures that are obtained from these values Eq. (3) (Zapata-Mina et al. 2023; Ayd 2021):

3 $$\gamma =1338-6\times {10}^{-5}{T}_{cc}+1\times {10}^{-8}{T}_{cc}^{2}$$

The temperature in the combustion chamber is deduced from Eq. (4) and is denoted by the symbol ${T}_{cc}$:

4 $${T}_{CC }=\frac{P.V.{T}_{air}}{{P}_{air} {V}_{ic}}$$

*T*_*air*_* P*_*air*_* V*_*ic*_ in Eq. (4), where *T*_*air*_, *Pair* is the air intake temperature, pressure and *V*_*ic*_ is the volume of the air that was being compressed when the engine was at its top dead centre.

### Uncertainty

The assessment of uncertainty involved in instrumentation is important in order to find overall error in the experimentation. In the experiments that were carried out for this research, the combined standard uncertainty *y* is assessed using Eq. (5) (Bitire and Jen 2023, Altun et al. 2023):

5 $$y =\sqrt{{(y}_{TS}^{2}}+{y}_{PS}^{2}+{y}_{SS}^{2}+{y}_{E}^{2}+{y}_{LC}^{2}+{y}_{B}^{2}+{y}_{CO}^{2}+{y}_{{CO}_{2}}^{2}+{y}_{HC}^{2}+{y}_{{O}_{2}}^{2}+{y}_{{NO}_{X}}^{2}+{y}_{BTE}^{2}+{y}_{BSC}^{2}+{y}_{EGT}^{2})$$

Table 4 outlines the uncertainty for various instruments. The combined uncertainty was found to $\pm$ 3.57, which is well within permissible limit.

**Table 4:** The *y* of the instrument

Instruments	Uncertainty (%)
Pressure sensor	± 0.5
Encoder	± 0.2
Speed sensor	± 1.0
Temperature sensor	± 0.15
Burette for fuel measurement	± 1.0
Load cell	± 0.2
${{\text{CO}}}_{2}$	± 1.0
${{\text{O}}}_{2}$	± 0.3
CO	± 0.3
${{\text{NO}}}_{x}$	± 0.5
HC	± 0.1
BTE	± 1.5
BSC	± 2.0
EGT	± 1.5

### Artificial neural network

**Fig. 3:**
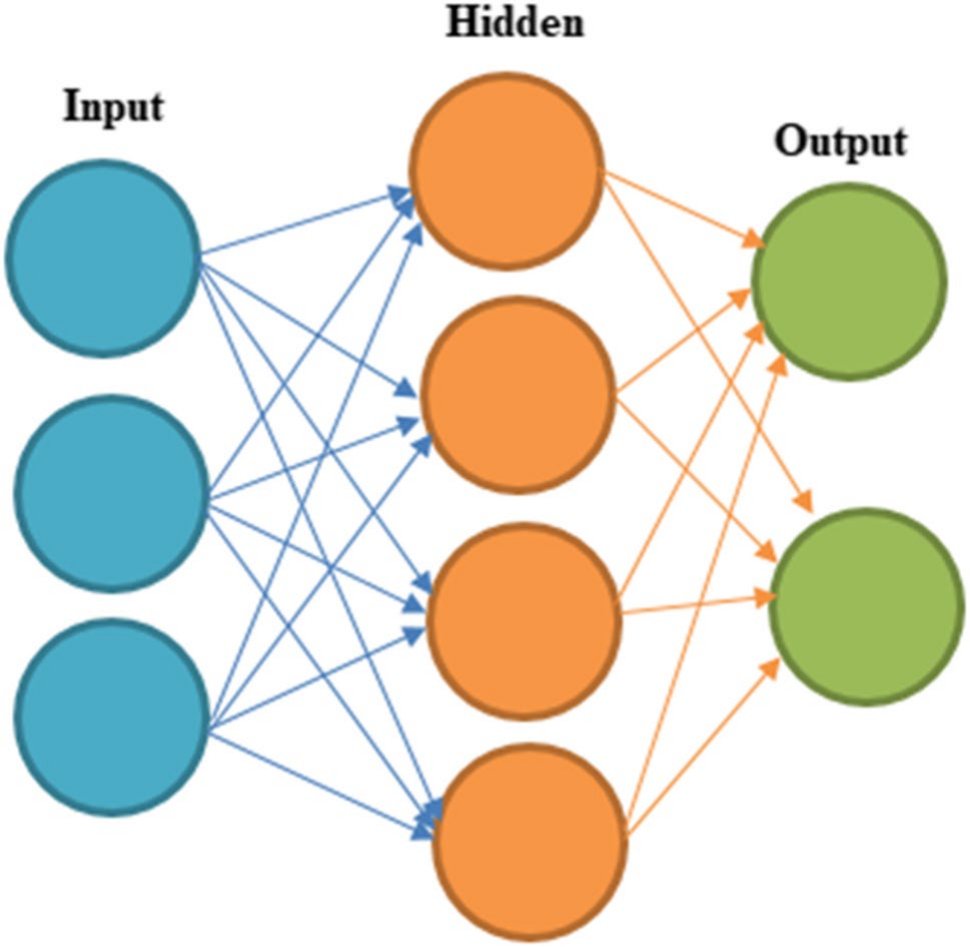
Structure of ANN model

There are special computers called ANN that can solve hard modelling problems that are not linear and are very complicated in a way that can be predicted. Instead of using an empirical calculation, the artificial neural network model learns from a large amount of input and output data. Remember that the activation function decides what the result is (Afzal et al. 2023; Elumalai et al. 2022; Veza et al. 2022). The input value and the cut off value are both shown in the functions output. The input that is connected to a node is given a weight that shows how strong or important it is. Adding an offset element also changes the strength of the input, which makes it easier for the activation function to be transferred (Oguz et al. 2010). The architecture of the ANN is shown in Fig. 3.

**Fig. 4:**
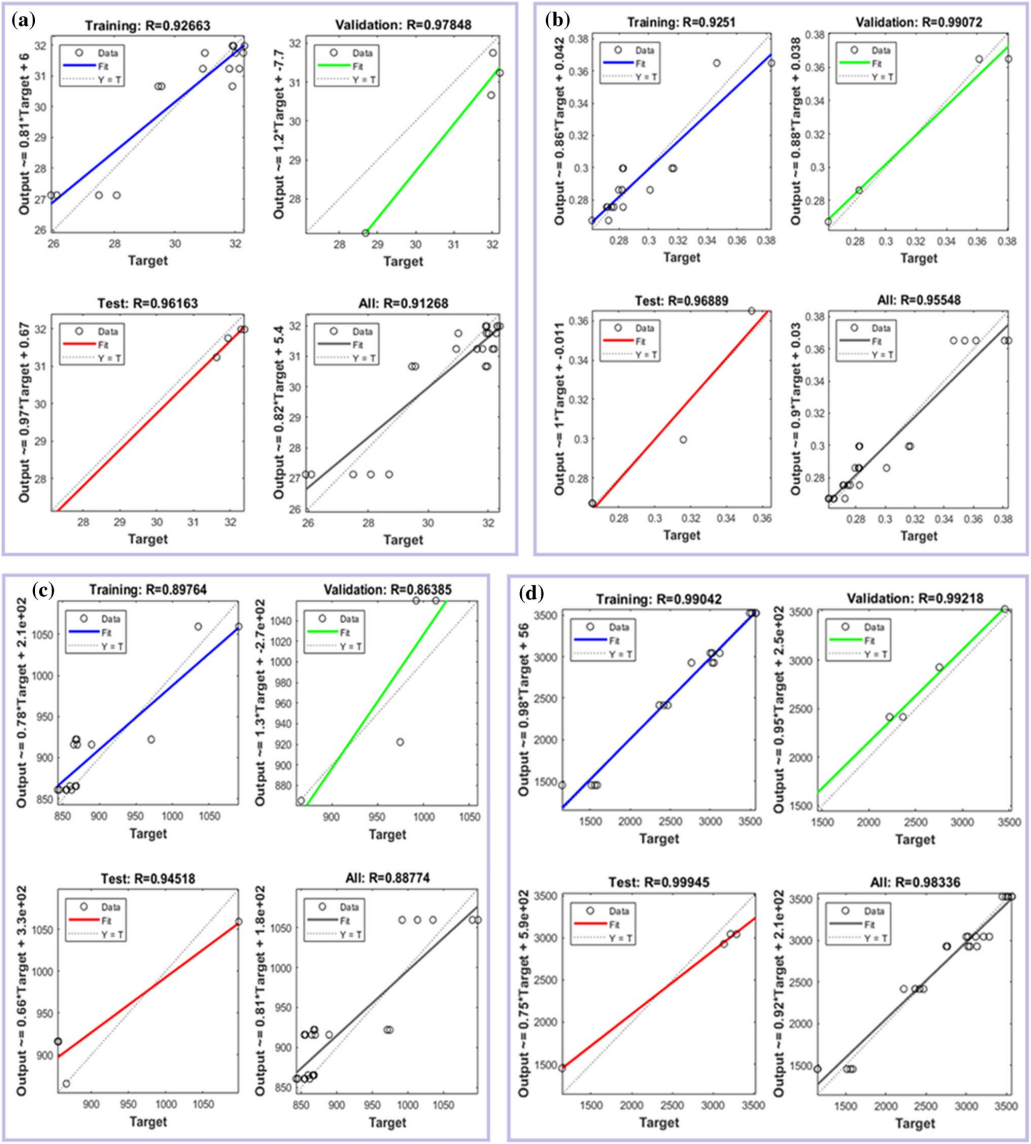
Training, validation, test and regression **a** BTE, **b** SFC, **c** CO_2_ emission and **d** ${{\text{NO}}}_{x}$ emission

ANNs are complicated modelling methods that copy neurons in the brain. Thanks to ANN, it is easier to find relationships between output and input factors that are not straight lines. Another good thing about ANN is that it can be quickly trained in a number of different ways. As part of this study, ANN was used to predict the predicted rise in efficiency. Figure 3 shows how the network is built. It has hidden layers that deal with input and output factors. Traindx (feed-forward back propagation) (Afzal et al. 2021; Mokashi et al. 2021), learngdm (adaptive learning function) and tansig (transfer function) were used on very large datasets to teach the ANN model. Mean squared error (MSE) was used to measure the results. The entire data was divided in training set, testing set and validation set in the ratio of 70:15:15. The data was divided randomly to ensure mix of all kind of data range for various aforementioned datasets (Khatri et al. 2023; Thodda et al. 2023; Seo and Park 2023). The training process for the ANN model was finished after 1000 epoch. The model is now reporting a gradient of ${10}^{-5}$ and an error rate of 0 (Prakash and Dhanasekaran 2022; 2023). In addition, the validation data were also checked over a thousand times. As shown in Fig. 4 a, b, c and d, the learning network model was trained, validated, tested and looked at for regression. The results shown in Fig. 4 show that all training data, validation data and testing data show good fitting with *R*^2^ value around 0.9.

## Results and discussion

### Brake-specific fuel consumption

**Fig. 5:**
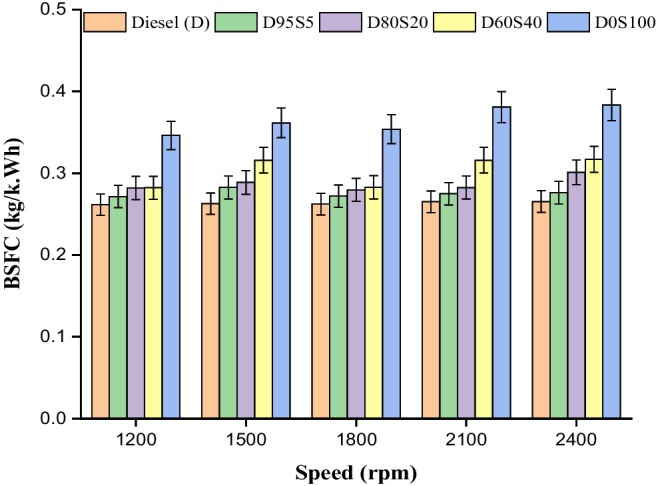
BSC with speed for blends

The change in the brake specific fuel consumption (BSFC) that occurs at various engine speeds can be seen in Fig. 5. Bio-diesel blends have a greater viscosity and a lower calorific value than diesel; all of them have shown a little improvement in their BSFC ratings when compared to diesel. In addition, the addition of biofuel blends causes an increase in the delay caused by the burning of molecules of extra oxygen. The higher cylinder temperatures that result from an increase in combustion delay lead to an increase in the amount of fuel that is used. This work investigated the least BSFC for slow-speed circumstances (Sharma et al. 2023; Rajak et al. 2021). Increasing the engine’s rotational speed results in an increase in the amount of gasoline used overall. When it comes to figuring out how significant a biodiesel mix is, BSFC is one of the most important parameters to consider. A greater consumption of fuel results in higher running expenses and causes for worry from an economic point of view. Regardless of the speeds at which the engines were operating, greater BSFC rates were observed for all of the mixes with the exception of D95S5 and D80S20. It was claimed that the minimal BSFC occurred at 1200 rpm, while the highest BSFC occurred at 2400 rpm. BSFC values for diesel, D95S5, D80S20, D60S40 and D0S100 at 2400 rpm with maximum capacity load were 0.265 kg/kWh, 0.276 kg/kWh, 0.301 kg/kWh and 0.317 kg/kWh, respectively. D0S100 had the highest BSFC value, at 0.38 kg/kWh.

### BTE

**Fig. 6:**
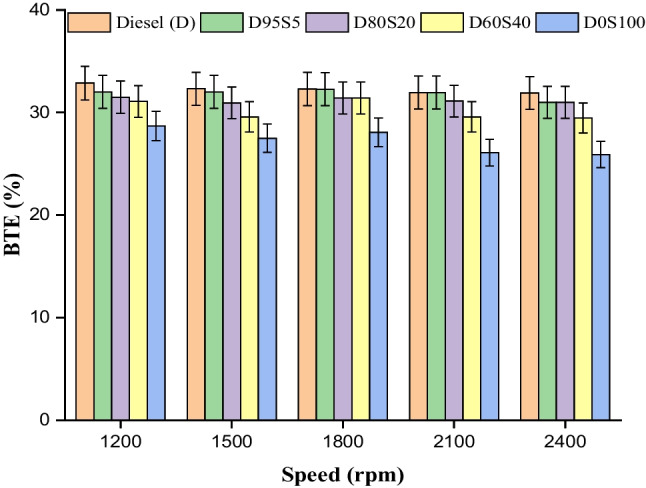
BTE with speed for blends

The efficiency of the various biodiesel blend in the diesel engine may be measured using Brake thermal efficiency (BTE), which is one of the most accurate criteria available to assess engine performance. In this test, the BTE was determined by comparing the load conditions of the engine to a number of different fuel mixes. In general, the thermal efficiency (BTE) of the fuel depends on two important characteristics, namely, the calorific value and the cetane number (Lalsangi et al. 2023). Because of diesel’s greater viscosity, it has a higher cetane number when compared to all biodiesel mixes. The pace at which something burns is dependent on a number of different physicochemical parameters, one of the most important of which is density. The change in BTE is shown here by figure across all of the different speeds. Because of its relatively high viscosity, biodiesel has a low thermal efficiency when compared to other fuels. Since of this, straight blends are not allowed to be used in diesel engines since they result in inefficient operation. In this portion of the test, several diesel mixes (D95S5, D80S20, D60S40 and D0S100) are put through their paces at a variety of engine speeds while operating at full capacity. Because of the increased combustion rates that occur at 1500 rpm, the thermal efficiency of the engine drops as the speed of the engine rises. On the other hand, the BTE of the mixes increases when the engine loading circumstances are greater. Maximum BTE values for diesel, D95S5, D80S20, D60S40 and D0S100 blends are as follows: 32.2%, 32.1%, 32.2%, 31.9% and 28.1%, respectively. The maximum recorded performance was achieved by a mix consisting of 20% biodiesel and 80% diesel. Because of the contribution of diesel as well as the low concentration of the blends, D80S20 fared the best out of all of the blends. In most cases, a significant reduction in thermal efficiency may be expected as the concentration of the mixes is increased. The viscosity of the fuel and the thermal efficiency of the system have characteristics that are diametrically opposed to one another (Fig. 6).

### VE

**Fig. 7:**
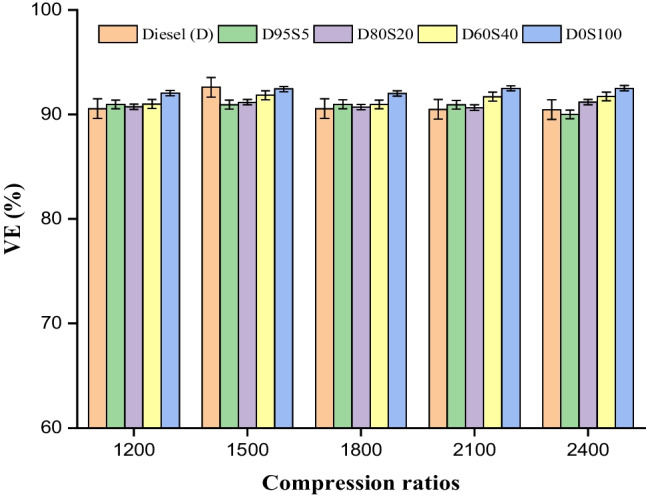
VE with speed for blends

Volumetric efficiency (VE) is one of the greatest methods to determine how successful the mixes are in a diesel engine, and it is also one of the ways. In this test, the TE was determined by comparing the load conditions of the engine to a number of different fuel mixes. Calorific value and cetane number are the main criteria determining fuel VE. Because of diesel’s higher viscosity compared to other types of biofuel mixes, it has a lower cetane number. The pace at which something burns is dependent on a number of different physicochemical parameters, one of the most important of which is density. The change in VE is shown here by figure across all of the different speeds. The increased oxygen rates contributed to the biofuel’s better volumetric efficiency when compared to conventional fuels. Since of this, the diesel engine does not make use of direct blends since doing so would result in improved efficiency. Here, the test mixes for diesel were D95S5, D80S20, D60S40 and D0S100. The results of these tests were 90.4%, 90.0%, 91.2%, 91.7% and 92.4%, respectively. These results were acquired by running the engines at different speeds while operating at full capacity (Fig. 7).

### EGT

**Fig. 8:**
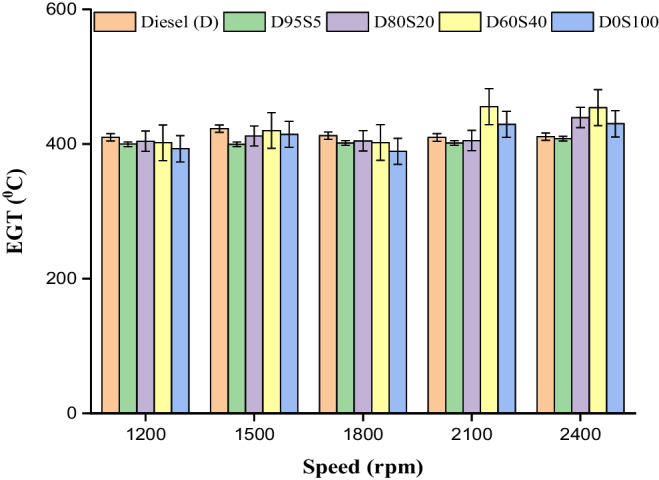
EGT with speed for blends

Through the use of a k-type thermocouple, the temperature of the exhaust gas may be utilised to monitor the temperature of the exhaust from an internal combustion engine. Exhaust gas temperature (EGT) has been observed such that changes in the air–fuel mixture may be seen. Figure 4 illustrates how the temperature of the gas behaves under a variety of different speed situations. Because biofuel contains more ester molecules than conventional gasoline, it ignites more quickly during the exhaust strokes. The EGT has been raised in comparison to the conditions imposed by the engine speed (Rajak et al. 2021; Lalsangi et al. 2023; Rajak et al. 2019). All biofuel blends exhibited an increase in the AET; however, the increases were between 5 and 20% lower when compared to diesel. On the other hand, the inclusion of SME resulted in a reduction in the AET of up to 20%. The temperatures that were recorded by the AET for the diesel mixes D95S5, D80S20, D60S40 and D0S100 at 2400 rpm were as follows: 411 °C, 408 °C, 439.5 °C, 454 °C and 430.3 °C, respectively. When compared to diesel, D80S20, D60S40 and D0S100, the EGT for the D95S5 was shown to have a significant decrease (Fig. 8).

### MCP

**Fig. 9:**
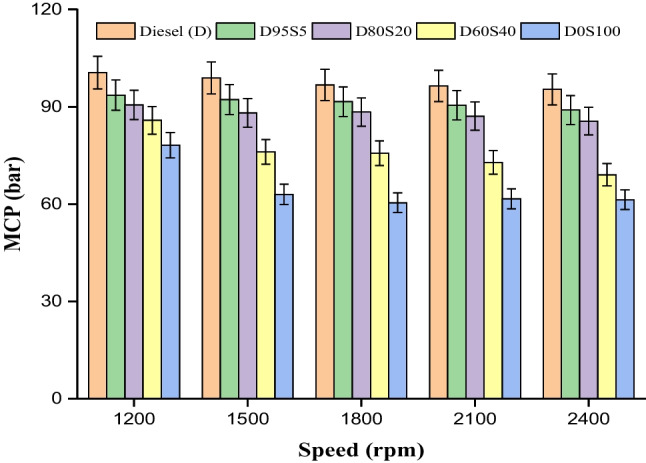
CMP with speed for blends

The pressure that builds up in an engine’s cylinder during a power stroke is called “in-cylinder pressure.” This pressure equals the observed fuel burn rate for effort. The fuel–air mixture, ignition delay time, premixed combustion fuel burning, atomization, viscosity, evaporation and thermal energy also affected in-cylinder pressure (Gavaskar et al. 2023; Musthafa et al. 2023). Compared to the figure, complete fuel mixes and basic diesel fuel will have varied cylinder maximum cylinder pressure (MCP) and engine rpm. The figure showed that diesel fuel, at a pressure of 122 bar, had a wider in-cylinder pressure range than other fuel blends. Next, diesel fuel blends D95S5, D80S20, D60S40 and D0S100 had a lower range of cylinder pressure rise when the load was bigger and the engine rotated at 2400 RPM. Then, the test fuel blends D95S5, D80S20, D60S40 and D0S100 had pressures of 115 bar, 110 bar, 99 bar and 81.5 bar. Due to heat value in biodiesel fuel, higher premixed combustion burning, a longer ignition delay period, dual fuel operation and higher energy release and combustion rate, fuel blends D95S5 and D80S20 had a range closer to diesel fuel. This happened for several reasons. The cylinder pressure of the mix D95S5 was lower than that of the premium diesel at higher load levels. As a consequence of this, the cylinder pressure of the mix D95S5 reduced by 5.7% in comparison to diesel fuel. At peak load circumstances with 2400 revolutions per minute, the cylinder pressure loss for the blends D80S20 and D60S40 was reduced by 9.8% and 18.0%, respectively, as compared to diesel. In a dual-fuel engine, rapid combustion generates more heat, which in turn boosts the cylinder pressure and temperature (Zandie et al. 2023; Abishek et al. 2024). At lower loads, the fuel mixture has a tendency to be rich, which leads to incomplete combustion. This happens due to the gas being supplied at reduced pressures. When operating at reduced loads, some fuel sample remains unburned, leading to concerns about emissions (Mani et al. 2011; Zhang et al. 2020) (Fig. 9).

### ROPR

**Fig. 10:**
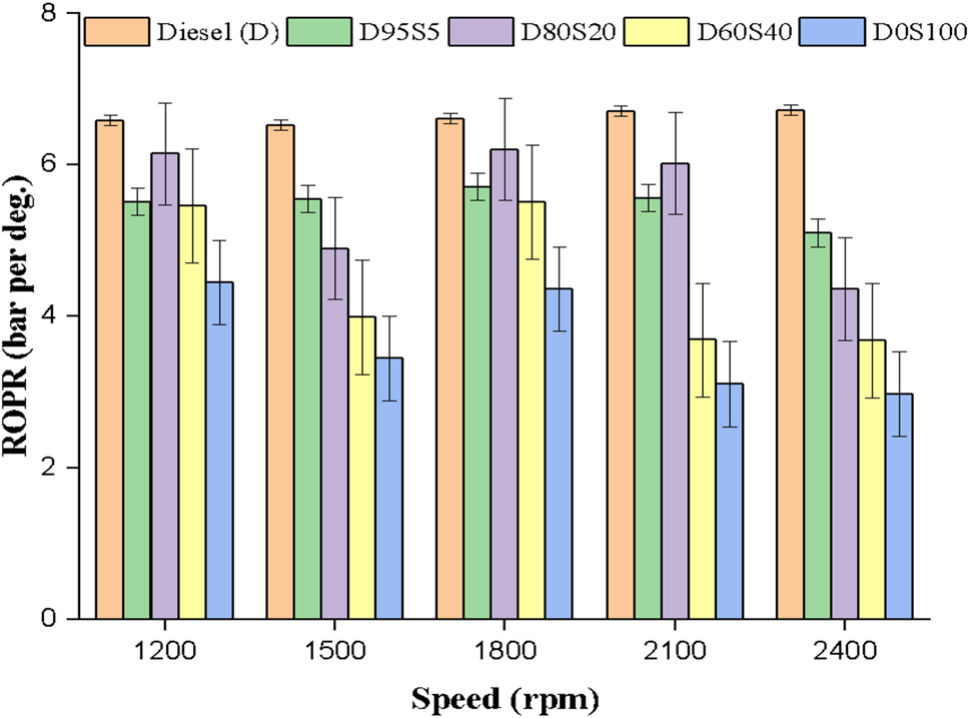
ROPR with speed for blends

The rate of pressure rise (ROPR) is a crucial factor in evaluating the efficiency of a biofuel blend, and incorporating the soybean biofuel into diesel engine together with diesel fuel has significantly reduced the ROPR. ROPR reduced when 5, 20 and 40% soybean was added to diesel fuel at all the engine conditions tested in the current study. The reduction in ROPR of 14% and 7.4% for 5% and 20% soybean biofuel blends may be due to the higher oxygen content and early burring of soybean biofuel (Fig. 10).

### Smoke emission

**Fig. 11:**
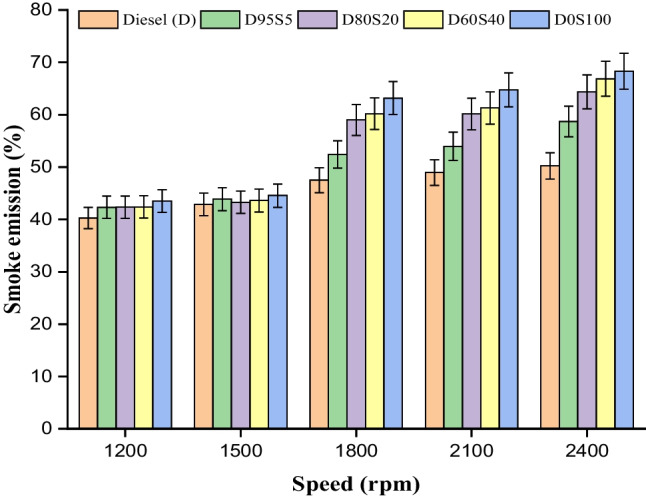
Smoke with speed for blends

The smoke emission is an important factor in evaluating a biofuel blend, and using S biofuel in the diesel engine with diesel fuel has greatly reduced smoke emission, improving combustion kinetics (Afzal et al. 2023). The current study shows a significant reduction in smoke emissions compared to Santhosh et al., who found that adding diesel, biodiesel and ethanol to diesel fuel increases emissions and decreases CI engine performance (Effendy et al. 2021). Kandasamy et al. (2019) found that adding 20% ethanol to basic petrol (B5) significantly reduced smoke. Smoke forms from incomplete combustion in the fuel-rich zone at high temperature and pressure. More specifically, this happens at the fuel spray’s centre. Oxygenates added to diesel fuel are often thought to oxygenate the pyrolysis zone of the burning diesel spray, lowering smoke (Kandasamy et al. 2019; Gowrishankar and Krishnasamy 2023). In the present study, smoke emission decreased with the addition of 5% and 20% S biofuel to diesel in all tested engine boundary conditions; however, smoke emission increased when the biofuel content exceeded 20%. For 5% and 20%, S fuel blend reduction in smoke emission was by 10% and 4%, respectively; this may be a result of biofuel’s higher oxygen content (Fig. 11).

### CO_2_ emission

The carbon dioxide (${{\text{CO}}}_{2}$) emission is an essential component in assessing the performance of a biofuel blend, and the utilisation of the S biofuel in the diesel engine with diesel fuel has slightly increased the ${{\text{CO}}}_{2}$ emission; thus, the utilisation of the S biofuel in the diesel is enhancing the combustion kinetics of the reaction (Jeyaseelan et al. 2023). In comparison to the findings of researches, which state that the addition of biofuel to diesel fuel causes an increase in emissions to a greater degree, which in turn increases the performance characteristics of a CI engine, the results of the current study demonstrate a significant improvement in the amount of CO_2_ emission that is emitted into the atmosphere (Fan et al. 2023). In the present study, the value of ${{\text{CO}}}_{2}$ emission was obtained to be 855.3 g/kWh for diesel, 867 g/kWh for D95S5, 869.7 g/kWh for D80S20, 974.4 g/kWh for D60S40 and 1098 g/kWh for D0S100; CO_2_ emission increased with the addition of 5% 20% and 40% S biofuel to diesel in all tested engine boundary conditions. Five per cent and 20% S fuel blend increased in ${{\text{CO}}}_{2}$ emission by 1.3% and 1.6%, respectively; this may be a result of biofuel higher oxygen content.

### ${{\text{NO}}}_{{\text{x}}}$ emission

**Fig. 12:**
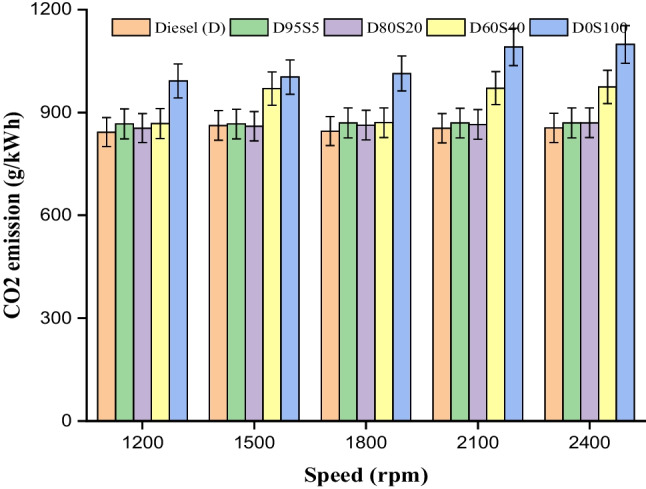
${{\text{CO}}}_{2}$ exhaust with speed for blends

Figure 12 shows that varying percentages of soybean biofuel with diesel fuel as D95S5, D80S20 and D60S40 blends had low nitrogen oxide (${{\text{NO}}}_{x}$) emissions at 1200, 1500, 1800, 2100 and 2400 rpm with the engine at full capacity. One of the primary contributors to the production of ${{\text{NO}}}_{x}$ is the presence of high temperatures inside the cylinder. In a similar fashion, an excessive amount of molecular oxygen in the fuel may also encourage the generation of ${{\text{NO}}}_{x}$. This occurs because the molecular oxygen in the fuel reacts with nitrogen during the combustion process to produce ${{\text{NO}}}_{x}$ emissions in the exhaust.

**Fig. 13:**
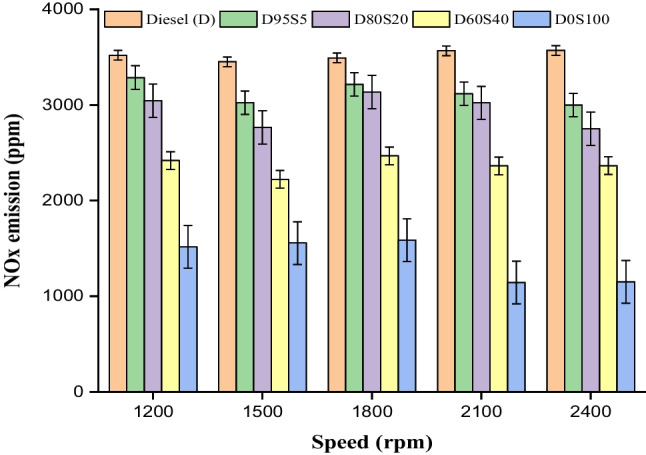
${{\text{NO}}}_{x}$ exhaust with speed for blends

This process is referred to as the Zeldovich reaction. Research conducted by Masera et al. in 2023 found that biofuel engines produce high levels of ${{\text{NO}}}_{x}$ emissions due to the presence of molecular oxygen. Low ${{\text{NO}}}_{x}$ emissions were recorded due to low-temperature combustion (Hoekman and Robbins 2012). The study found that the D95S5 and D80S20 fuel blends, which contain 5% and 20% of soybean biofuel, respectively, produced very low levels of ${{\text{NO}}}_{x}$ emissions in all tested scenarios. At 2400 rpm, the D80S20 blend with 20% share of soybean biofuel produces lower ${{\text{NO}}}_{x}$ emissions than diesel fuel up to 22.2%. Using oxygenated soybean enhances fuel properties, resulting in decreased residence time and reduces delay duration. Soybean biofuel helps produce a lower adiabatic flam temperature, minimizes temperature rise in the surrounding area and promotes low-temperature combustion (Mirhashemi and Sadrnia 2020; Kalyani et al. 2023; Masera and Hossain 2023). As a consequence of this, the additive in the D80S20 mix demonstrates significantly reduced levels of ${{\text{NO}}}_{x}$ emissions. The significant downfall in ${{\text{NO}}}_{x}$ emission of D80S20 blend might be credited to elevated oxygen level and high moisture level of biodiesel. An elevated moisture percentage in biodiesel leads reduction in chamber temperature which in turn limits the ${{\text{NO}}}_{x}$ formation (Sarıdemir and Agbulut 2022; Agbulut et al. 2020; Prakash and Dhanasekaran 2019) (Fig. 13).

## Conclusion

The primary experimental results from the present investigation are outlined below.

- Diesel fuel operated at 1500 rpm exhibits a better brake thermal efficiency of 32.4%, which is 1.5% higher than diesel fuel run at 2400 rpm. Soybean biofuel usage led to a small decrease in brake thermal efficiency under all investigated conditions. The brake thermal efficiency of the D80S20 engine operating at various speeds exhibits slightly lower value compared to diesel across all rpm circumstances.
- Brake-specific fuel consumption increases by 1.8% for diesel fuel running at 2400 rpm compared to 1500 rpm. Additionally, using a blend of 20% soybean biofuel and diesel fuel results in a 7.1% increase in brake-specific fuel consumption at 1500 rpm.
- The combustion characteristics, such as cylinder pressure, were determined for diesel (D) and D80S0 fuel operating at 1500 rpm with pressure of 98 bar and 92.2 bar, at a compression ratio of 18 and full load. ROPR reductions of 14% and 7.4%, respectively, for 5% and 20% S fuel blends may be the consequence of biofuel higher oxygen content and early combustion.
- At 2400 rpm with maximum load, the ${{\text{CO}}}_{2}$ emission value was determined to be 855.3 g/kWh for diesel, 867 g/kWh for D95S5, 869.7 g/kWh for D80S20, 974.4 g/kWh for D60S40 and 1098 g/kWh for D0S100. ${{\text{CO}}}_{2}$ emission increased with the addition of 5%, 20% and 40% S biofuel to diesel in all tested engine boundary conditions. ${{\text{CO}}}_{2}$ emissions rose 1.3% and 1.5% for 5% and 20% S blends, respectively.
- According to this study, the D95S5 and D80S20 mixtures containing 5% and 20% biofuel, respectively, had minimal ${{\text{NO}}}_{x}$ emissions under all examined conditions. At 2400 revolutions per minute, the D80S20 blend with a 20% blend of biofuel produces lower ${{\text{NO}}}_{x}$ emissions than diesel fuel.

To investigate the impact of various types of nanoparticles added to the D80S20 blend, optimize engine parameters with the use of response surface methodology, Taguchi method and artificial neural networks.

## Data Availability

Data available on request.

## Nomenclature

AI: Artificial intelligence
ANN: Artificial neural network
AET: Average exhaust gas temperature
B: Burette for fuel measurement
BSC: Brake specific fuel consumption
BSN: Bosch smoke number
b TDC: Before top dead centre
BTE: Brake thermal efficiency
CI: Compression ignition engine
CMP: Cylinder maximum pressure
CMT: Cylinder maximum temperature
CO: Carbon monoxides
CO_2_: Carbon dioxide
CR: Compression ratio
D: Diesel fuel
D95S5: Diesel fuel 95% and S fuel 5%
D80S20: Diesel fuel 80% and S fuel 20%
D60D40: Diesel fuel 60% and S fuel 40%
D0S100: Diesel fuel 0% and S fuel 100%
E: Encoder
EGT: Exhaust gas temperature
HC: Hydrocarbon
IC: Internal combustion
LC: Load cell
${{\text{NO}}}_{x}$: Oxides of nitrogen
O_2_: Oxygen
POID: Period of ignition delay
PM: Particulate matter
PS: Pressure sensor
ROPR: Rise of pressure rate
S: Soybean oil methyl ester
SS: Speed sensor
TS: Temperature sensor
VE: Volumetric efficiency
y: Uncertainty
